# Association between Self-Reported Smoking and Hemoglobin A1c in a Korean Population without Diabetes: The 2011–2012 Korean National Health and Nutrition Examination Survey

**DOI:** 10.1371/journal.pone.0126746

**Published:** 2015-05-26

**Authors:** Jae Won Hong, Cheol Ryong Ku, Jung Hyun Noh, Kyung Soo Ko, Byoung Doo Rhee, Dong-Jun Kim

**Affiliations:** 1 Department of Internal Medicine, Ilsan-Paik Hospital, College of Medicine, Inje University, Koyang, Gyeonggi-do, South Korea; 2 Endocrinology, Yonsei University College of Medicine, Seoul, South Korea; 3 Department of Internal Medicine, Sanggye Paik Hospital, Cardiovascular and Metabolic Disease Center, College of Medicine, Inje University, Seoul, Republic of Korea; University of Arkansas for Medical Sciences, UNITED STATES

## Abstract

**Background:**

Several Western studies have revealed that among non-diabetics, glycosylated hemoglobin A1c (HbA1c) levels are higher in smokers than non-smokers. While studies conducted in Western populations consistently support this association, a recent meta-analysis reported that studies carried out in non-Western populations, including studies of Chinese, Egyptian, and Japanese-Americans, did not detect any significant differences in HbA1c levels between smokers and non-smokers.

**Objectives:**

We assessed the association between smoking habits and HbA1c levels in the general Korean adult population using data from the Korean National Health and Nutrition Examination Survey (KNHANES) performed in 2011–2012.

**Methods:**

A total of 10,241 participants (weighted n=33,946,561 including 16,769,320 men and 17,177,241 women) without diabetes were divided into four categories according to their smoking habits: never smokers (unweighted *n/* weighted *n*= 6,349/19,105,564), ex-smokers (unweighted *n/* weighted *n*= 1,912/6,207,144), current light smokers (<15 cigarettes per day, unweighted *n/* weighted *n*=1,205/5,130,073), and current heavy smokers (≥15 cigarettes per day, unweighted *n/* weighted *n*=775/3,503,781).

**Results:**

In age- and gender-adjusted comparisons, the HbA1c levels of each group were 5.52 ± 0.01% in non-smokers, 5.49 ± 0.01% in ex-smokers, 5.53 ± 0.01% in light smokers, and 5.61 ± 0.02% in heavy smokers. HbA1c levels were significantly higher in light smokers than in ex-smokers (*p* = 0.033), and in heavy smokers compared with light smokers (*p* < 0.001). The significant differences remained after adjusting for age, gender, fasting plasma glucose, heavy alcohol drinking, hematocrit, college graduation, and waist circumference. Linear regression analyses for HbA1c using the above-mentioned variables as covariates revealed that a significant association between current smoking and HbA1c (coefficient 0.021, 95% CI 0.003–0.039, *p* = 0.019).

**Conclusions:**

Current smoking was independently associated with higher HbA1c levels in a cigarette exposure-dependent manner in a representative population of Korean non-diabetic adults. In this study, we have observed an association between smoking status and HbA1c levels in non-diabetics drawn from a non-Western population, consistent with previous findings in Western populations.

## Introduction

Glycosylated hemoglobin A1c (HbA1c) is a marker for long-term glucose control and provides an index of overall glycemic exposure and risk for long-term complications in patients with diabetes. It is used to guide management and adjust treatments for diabetic individuals. In addition to monitoring glycemic control, HbA1c is also used to diagnose diabetes and identify individuals at high risk of developing diabetes [1]. However, HbA1c levels can be influenced by various factors including age, ethnicity, conditions that alter red cell turnover, socioeconomic factors, and glucose homeostasis [1–6].

Cigarette smoking is a major public health problem and a well-known risk factor for cardiovascular disease and several malignancies. Many studies have shown that cigarette smoking is also associated with an increased risk of diabetes and insulin resistance [7–11]. Furthermore, several studies have reported that current smokers exhibit higher HbA1c levels than non-smokers, even in populations without diabetes [9,12–16].

Recently, Soraya *et al*. analyzed the difference in mean HbA1c levels between current smokers and never-smokers. Their meta-analysis included individuals from 14 countries and suggested that HbA1c levels are higher in smokers than in non-smokers without known diabetes.[17] Therein, higher HbA1c levels in current smokers were consitently reported in 10 out of 14 Western population studies, including those from Australia, France, the United States, the Netherlands, and Denmark. However, in Chinese, Egyptian, Kenyan and Japanese-American populations, differences in HbA1c levels were not detected; current smokers even showed lower HbA1c levels. Therefore, race/ethnicity, or western lifestyle may modify the association between smoking and HbA1c levels. Nevertheless, few studies have investigated the association between smoking status and HbA1c levels in large study samples, particularly in non-Western populations.

In the current study, we performed a cross-sectional analysis to investigate the association between HbA1c and smoking habits in a racially homogeneous Korean adult population.

## Method

### Study population and data collection

This study used data from the 2011–2012 Korea National Health and Nutrition Examination Survey (KNHANES), a cross-sectional and nationally representative survey conducted by the Korean Center for Disease Control for Health Statistics. The KNHANES has been performed periodically since 1998 to assess the health and nutritional status of the civilian, non-institutionalized Korean population. Participants were selected using proportional allocation systematic sampling with multistage stratification. A standardized interview was conducted in the homes of the participants to collect information regarding demographic variables, family history, medical history, medications used, and a variety of other health-related variables. The health interview included well-established questions to determine the demographic and socioeconomic characteristics of the subjects, including questions concerning age, education level, occupation, income, marital status, smoking habits, alcohol consumption, exercise, previous and current diseases, and family disease history. Smoking status was divided into four categories: never smoker, ex-smoker, current light smoker (<15 cigarettes per day), and current heavy smoker (≥15 cigarettes per day).

Subjects were asked whether they exercised with an intensity that caused slight breathing difficulty and sweating. Subjects who exercised regularly at moderate intensity were asked about the frequency at which they exercised per week and the length of time per exercise session. Regular exercise was defined as exercising five or more times per week. Alcohol consumption was assessed by questioning the subjects about their drinking behavior during the month prior to the interview. Heavy alcohol drinking was categorized as drinking four or more times per week. Hypertension was defined as a systolic blood pressure (BP) ≥140 mmHg, diastolic BP ≥90 mmHg, or the use of antihypertensive medications, irrespective of blood pressure. Diabetes was defined as a fasting plasma glucose ≥ 126 mg/dL (7.0 mmol/l), current use of anti-diabetes medications, or a previous diagnosis of diabetes by a physician. In this study, the definition of diabetes did not include HbA1c level criteria. Obesity was defined as a body mass index (BMI) ≥25 kg/m^2^ according to the Asia-Pacific obesity classification [18].

Height and weight were obtained using standardized techniques and equipment. Height was measured to the nearest 0.1 cm using a portable stadiometer (Seriter, Bismarck, ND, USA). Weight was measured to the nearest 0.1 kg using a Giant-150N calibrated balance-beam scale (Hana, Seoul, Korea). BMI was calculated by dividing weight by the square of the height (kg/m^2^). Systolic and diastolic BP were measured by standard methods using a sphygmomanometer and with the patient in the sitting position. Three measurements were made for all subjects at 5-min intervals, and the mean of the second and third measurements was used in the analysis.

### Laboratory methods

Blood samples were collected in the morning after fasting for at least 8 h. Fasting plasma glucose (FPG), total cholesterol, triglycerides (TG), low-density lipoprotein cholesterol (LDL-C), and serum creatinine levels were measured using a Hitachi Automatic Analyzer 7600 (Hitachi, Tokyo, Japan). HbA1c was measured using high performance liquid chromatography (HLC-723G7, Tosoh, Tokyo, Japan). The detailed methods for comparing and verifying the validity and reliability of each survey were described previously [19].

### Ethics statement

The institutional review board of Ilsan Paik Hospital, Republic of Korea (IB-2-1404-016) approved this study. After approval of the study proposal, the KNHANES dataset was made available at the request of the investigator. Since the dataset did not include any personal information and participant consent had already been given for the KNHANES, our study was exempt from participant consent requirements.

### Statistical analyses

**Fig 1:**
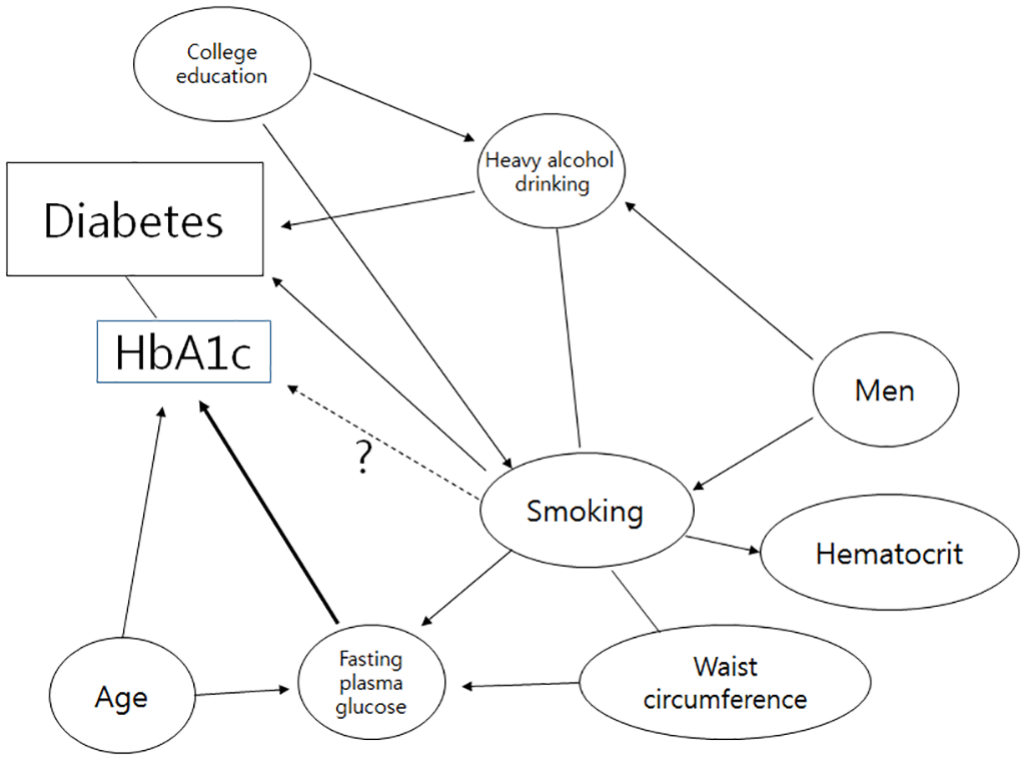
DAG summarizes the established and hypothesized causal relationships among variables under study.

Participants in the Korean NHANES were selected for participation based upon a stratified, multi-stage probability-sampling design. Weights for each respondent, representing the inverse of their sampling probability, were provided by the Korean Center for Disease Control and have been used in most analyses presented in order to produce estimates representative of the non-institutionalized Korean civilian population. However, linear models used to identify statistically significant determinants of HbA1C level were not weighted. Statistical analyses were performed using SPSS software (ver. 21.0 for Windows; SPSS, Chicago, IL, USA) for all analyses. To consider relationships among the study variables, we have drawn a direct acyclic diagram (DAG) based on the previous research findings or known facts. (Fig 1) To compare demographic and clinical characteristics among groups according to smoking habits, we evaluated age by ANOVA (analysis of variance) and the percentage of males by the χ^2^-test. ANCOVA (analysis of covariance) with Bonferroni *post-hoc* test was used to adjust for age and gender (Table 1). General linear models were used to assess weighted HbA1c levels according to smoking habits before and after adjustment for confounders (Table 2). Age (year), sex (men/women), and FPG (mmol/l) were adjusted in Model1.

**Table 1:** Weighted, age- and sex-adjusted demographic and clinical characteristics by smoking status for Korean adults without diabetes, aged 19 years and older (2011–2012 KNHANES).

	Never smoker	Ex-smoker	Current smoker (<15 cigarettes/day)	Current smoker (≥ 15 cigarettes/day)	P
Number (unweighted/weighted)	6349/19105564	1912/6207144	1205/5130073	775/3503781	
Age (years)	44.5 ± 0.3	47.8 ± 0.5	38.9 ± 0.5	42.7 ± 0.5	<0.001
Women (%)	78.0 ± 0.8	16.0 ± 0.9	22.0 ± 1.2	3.0 ± 0.6	<0.001
College graduation (%)	32.9 ± 1.1	36.3 ± 1.6	30.6 ± 1.8	27.5 ± 2.1	0.022
Heavy alcohol drinking (%)	2.4 ± 0.3	10.6 ± 1.0	9.3 ± 1.0	19.5 ± 1.8	<0.001
Regular exercise (%)	7.4 ± 0.5	8.2 ± 1.0	7.7 ± 1.0	6.5 ± 1.2	1.000
Waist circumference (cm)	80.1 ± 0.2	80.7 ± 0.3	80.6 ± 0.4	82.6 ± 0.5	0.006
BMI (kg/m^2^)	23.5 ± 0.1	23.6 ± 0.1	23.6 ± 0.2	24.2 ± 0.2	0.014
Obesity (%)	29.7 ± 0.9	30.9 ± 1.4	28.4 ± 1.8	41.2 ±2.4	<0.001
Systolic BP (mmHg)	117.0 ± 0.3	115.9 ± 0.4	116.2 ± 0.5	116.6 ±0.6	0.162
Diastolic BP (mmHg)	76.0 ± 0.2	76.0 ± 0.3	75.3 ± 0.4	77.3 ±0.5	0.003
Anti-hypertensive drug (%)	10.7 ± 0.4	13.8 ± 0.9	11.9 ± 0.9	8.4 ± 1.0	0.005
Hypertension (%)	21.2 ± 0.7	25.2 ± 1.2	23.2 ± 1.3	24.4 ± 1.9	0.015
FPG (mmol/l)	5.12 ± 0.01	5.16 ± 0.02	5.09 ± 0.02	5.20 ± 0.03	<0.001
HbA1c (%)	5.52 ± 0.01	5.49 ± 0.01	5.53 ± 0.01	5.61 ± 0.02	<0.001
HbA1c (mmol/mol)	36.83 ± 0.11	36.50 ± 0.11	36.94 ± 0.11	37.81 ± 0.22	<0.001
Impaired fasting glucose (%)	19.0 ± 0.8	21.0 ± 1.2	17.0 ± 1.3	26.0 ± 2.0	0.001
Serum LDL-C (mg/dl)	115.2 ± 1.3	115.6 ± 1.8	114.4 ± 2.1	119.2 ± 2.7	0.462
Serum TG (mg/dl)	118.0 ± 1.9	126.7 ± 2.7	138.6 ± 4.1	181.0 ± 7.1	<0.001
Anti-lipid drug (%)	2.8 ± 0.2	3.8 ± 0.5	3.2 ± 0.4	3.3 ± 0.6	0.135
Serum Creatinine (mg/dl)	0.85 ± 0.01	0.84 ± 0.01	0.84 ± 0.01	0.84 ± 0.01	0.806
Hematocrit (%)	42.1 ± 0.1	41.9 ± 0.1	42.5 ± 0.1	43.3 ± 0.1	<0.001

Data are expressed as mean with SEM. The estimates for age and sex are not adjusted. BMI, body mass index; BP, blood pressure; FPG, fasting plasma glucose; LDL-C, low density lipoprotein-cholesterol; TG, triglyceride; Heavy alcohol drinking, ≥ ×4 alcoholic drinks/week. Regular exercise, ≥ ×5 exercise/week; Obesity, BMI ≥ 25 kg/m^2^ or more; Hypertension, systolic blood pressure ≥ 140 mmHg or diastolic blood pressure ≥90 mmHg or use of antihypertensive medications irrespective of BP;

**Table 2:** Weighted, multivariable-adjusted mean HbA1c (%) levels in Korean adults without diabetes by smoking status.

	Never smoker	Ex-smoker	Current smoker (<15 cigarettes/day)	Current smoker (≥ 15 cigarettes/day)	P
Number (unweighted/weighted)	6349/19105564	1912/6207144	1205/5130073	775/3503781	
Model 1					
NFG	5.44 ± 0.01	5.41 ± 0.01^a^	5.46 ± 0.01^b^	5.53 ± 0.02^C^	0.001
IFG	5.82 ± 0.02	5.79 ± 0.02	5.87 ± 0.03	5.87 ± 0.03	0.096
NFG+IFG	5.52 ± 0.01	5.49 ± 0.01^a^	5.54 ± 0.01^b^	5.59 ± 0.02^C^	0.001
Model 2					
NFG	5.44 ± 0.01	5.41 ± 0.01 ^a^	5.46 ± 0.01^b^	5.54 ± 0.02^C^	<0.001
IFG	5.81 ± 0.02	5.80 ± 0.02	5.87 ± 0.03 ^b^	5.89 ± 0.03	0.116
NFG+IFG	5.52 ± 0.01	5.49 ± 0.01 ^a^	5.54 ± 0.01^b^	5.60 ± 0.02^C^	<0.001

^a^, p< 0.05 vs. never-smoker;

^b^, p<0.01 vs. ex-smoker;

^c^, P<0.01 vs. current smoker (<15 cigarettes/day)

NFG, normal fasting glucose; IFG, impaired fasting glucose

Model 1, adjusted for age, sex, and fasting plasma glucose

Model 2, adjusted for heavy alcohol drinking, college graduation, hematocrit, waist circumference, and variables in Model 1

In Model 2, heavy alcohol drinking (≥ 4 alcoholic drinks/week/ <4 alcoholic drinks/week), college graduation (yes/no), hematocrit (%), and waist circumference (cm), as well as age, sex, and FPG, were adjusted for in the analysis.

Linear regression analysis for HbA1c was performed using age (years), gender (men/women), current smoking (yes/no), heavy alcohol drinking (≥ 4 alcoholic drinks/week/ <4 alcoholic drinks/week), college graduation (yes/no), waist circumference (cm), FPG (mmol/l), and hematocrit (%) levels as confounding variables (Table 3). There was no evidence that ANOVA and linear regression assumptions were violated. Logistic regression analysis was used to evaluate the odds ratios and 95% confidence intervals of ≥ 5.7% (the criteria of abnormal glucose regulation) and 6.1% HbA1c levels using age (by 10year increase), gender (men/ women), smoking status (never smoking/ ex-smoking/ current light smoking/current heavy smoking), heavy alcohol drinking (≥ 4 alcoholic drinks/week/ <4 alcoholic drinks/week), college graduation (yes/no), waist circumference (by 5cm increase), FPG (by 1 mmol/l), and hematocrit (by 2% increase) levels as confounding variables (Tables 4 and 5). The HbA1c cutoff value of 6.1% was selected, because a recent Korean study showed an HbA1c cutoff level of 6.1% to be the optimal corresponding value for diagnosing diabetes, along with the criteria of FPG ≥ 7.0 mmol/l and/or 2 hour plasma glucose ≥ 11.1 mmol/l upon a 75 gram oral glucose tolerance test (63.8% sensitivity and 88.1% specificity). An HbA1c threshold of 5.7% had reasonable sensitivity (48.6%) and specificity (65.7%) for identification of prediabetes.[20] All tests were two-sided, and *P* < 0.05 was considered statistically significant.

**Table 3:** Regression parameters from unweighted linear regression models to predict HbA1c levels in Korean adults without diabetes (n = 10,241).

	Coefficient (95% CI)	*P*
Age (year)	0.006 (0.006–0.0067)	<0.001
Women	0.060 (0.039–0.081)	<0.001
Current smoking (10cm increase)	0.021 (0.003–0.039)	0.019
Heavy alcohol drinking	-0.111 (-0.138- -0.084)	<0.001
College graduation	-0.018 (-0.032- -0.003)	0.019
Waist circumference	0.005 (0.004–0.005)	<0.001
Fasting plasma glucose (mmol/l)	0.283 (0.270–0296)	<0.001
Hematocrit (%)	-0.001 (-0.004–0.001)	0.176
R^2^	0.2	

**Table 4:** Estimates of association from weighted logistic regression models to predict the risks of HbA1c ≥ 5.7% or ≥ 6.1% in Korean adults without diabetes (n = 10,241).

	For HbA1c ≥ 5.7%		For HbA1c ≥ 6.1%	
	Odds ratio (95% CI)	p	Odds ratio (95% CI)	p
Age (10 year increase)	1.52 (1.45–1.59)	<0.001	1.65 (1.53–1.77)	<0.001
Men	0.88 (0.71–1.08)	0.203	0.68 (0.50–0.91)	0.009
Heavy alcohol drinking	0.48 (0.37–0.64)	<0.001	0.42 (0.28–0.63)	<0.001
Waist circumference (5cm increase)	1.15 (1.10–1.19)	<0.001	1.20 (1.14–1.26)	<0.001
College graduation	0.95 (0.82–1.10)	0.482	1.18 (0.91–1.53)	0.211
Smoking		<0.001		<0.001
Never smoking	reference		reference	
Ex-smoking	0.96 (0.78–1.17)	0.675	0.83 (0.62–1.12)	0.228
Current smoking				
<15 cigarettes/day	1.29 (1.04–1.60)	0.002	1.21 (0.82–1.78)	0.334
≥ 15 cigarettes /day	1.84 (1.41–2.41)	<0.001	2.36 (1.58–3.52)	<0.001
Hematocrit (2% increase)	0.94 (0.90–0.99)	0.011	0.93 (0.87–1.00)	0.041
Fasting plasma glucose (1mmol/l increase)	3.72 (3.29–4.21)	<0.001	8.66 (7.24–10.35)	<0.001

All odds ratios were from weighted analyses.

**Table 5:** Estimates of association from weighted logistic regression models to predict the risks of HbA1c ≥ 5.7% or ≥ 6.1% in Korean adults without diabetes after exclusion of subjects with HbA1c ≥6.5% (n = 10,026).

	For HbA1c ≥ 5.7%		For HbA1c ≥ 6.1%	
	Odds ratio (95% CI)	p	Odds ratio (95% CI)	p
Age (10 year increase)	1.51 (1.44–1.58)	<0.001	1.62 (1.50–1.75)	<0.001
Men	0.88 (0.72–1.09)	0.242	0.66 (0.48–0.91)	0.011
Heavy alcohol drinking	0.49 (0.37–0.65)	<0.001	0.43 (0.27–0.68)	<0.001
Waist circumference (5cm increase)	1.14 (1.10–1.18)	<0.001	1.15 (1.09–1.22)	<0.001
College graduation	0.94 (0.82–1.09)	0.423	1.14 (0.87–1.50)	0.334
Smoking		<0.001		<0.001
Never smoking	reference		reference	
Ex-smoking	0.97 (0.79–1.19)	0.766	0.93 (0.67–1.28)	0.644
Current smoking				
<15 cigarettes/day	1.29 (1.04–1.61)	0.023	1.25 (0.82–1.90)	0.297
≥ 15 cigarettes /day	1.83 (1.39–2.41)	<0.001	2.53 (1.61–4.00)	<0.001
Hematocrit (2% increase)	0.94 (0.90–0.99)	0.014	0.94 (0.87–1.01)	0.082
Fasting plasma glucose (1mmol/l increase)	3.44 (3.04–3.89)	<0.001	6.82 (5.60–8.30)	<0.001

All odds ratios were from weighted analyses.

## Results

### Demographics and clinical characteristics of the study population

The demographics and clinical characteristics of the study population are shown in Table 1. Among the 11,473 adult participants (≥19 years of age) who participated in health interviews and laboratory examinations in the 2011–2012 KNHANES, 1,232 diabetic subjects were excluded. The remaining 10,241 participants (weighted n = 33,946,561 including 16,769,320 men and 17,177,241 women) were divided into four categories according to their smoking habits: never smokers (unweighted *n/* weighted *n* = 6,349/19,105,564), ex-smokers (unweighted *n/* weighted *n* = 1,912/6,207,144), current light smokers (<15 cigarettes per day, unweighted *n/* weighted *n* = 1,205/5,130,073), and current heavy smokers (≥15 cigarettes per day, unweighted *n/* weighted *n* = 775/3,503,781).

The mean ages of the never smokers, ex-smokers, current light smokers, and current heavy smokers were 44.5 ± 0.3, 47.8 ± 0.5, 38.9 ± 0.5, and 42.7 ± 0.5 years, respectively. The percentages of female participants in each smoking group were 78.0, 16.0, 22.0, and 3.0%, respectively (*p* < 0.001). In age- and gender-adjusted comparisons, the current heavy smokers had a higher BMI, waist circumference, serum TG levels, and fasting plasma glucose levels than current light smokers, never smokers, and ex-smokers. Heavy alcohol drinking and obesity were more common among current heavy smokers than current light smokers, never smokers, and ex-smokers. There was no difference in waist circumference or BMI among never smokers, ex-smokers, and current light smokers. Heavy alcohol drinking was more common in ex-smokers and current light smokers than never smokers.

### Mean HbA1c levels according to smoking habits of the subjects with and without impaired FPG

After age- and gender-adjusted comparisons, mean HbA1c levels were higher among current smokers than never smokers and ex-smokers (5.56 ± 0.01, 5.52 ± 0.01, and 5.49 ± 0.01%, respectively). Among current smokers, heavy smokers also had a higher mean HbA1c level than light smokers (5.61 ± 0.02 vs. 5.53 ± 0.01%, *p* < 0.001). FPG levels were higher in current heavy smokers than current light smokers. However, there was no difference therein among never smokers, ex-smokers, and current light smokers (Table 1).

Age-, gender-, and FPG-adjusted HbA1c levels are shown in Table 2 (model 1). In all participants, including those with both normal and impaired FPG, mean HbA1c levels were slightly higher in current smokers than ex-smokers, even after adjusting for age, gender, and FPG. Furthermore, current heavy smokers demonstrated a higher mean HbA1c level than current light smokers, suggesting an exposure-dependent phenomenon. Subgroup analysis of individuals with normal FPG revealed similar results of a higher mean HbA1c level in current smokers than ex-smokers in a dose-dependent manner. Subgroup analysis of individuals with impaired FPG showed a similar trend, but without statistical significance.

A similar dose-dependent relationship between smoking exposure and HbA1c levels was identified in analyses using age, gender, FPG, heavy alcohol drinking, hematocrit, college graduation and waist circumference as covariates (Table 2, model 2; *p* < 0.001).

### Factors associated with elevated HbA1c levels

Linear regression analysis to identify independent factors associated with HbA1c levels were performed using age, gender, current smoking, heavy alcohol drinking, college education, waist circumference, FPG, and hematocrit levels as confounding variables. In this model, current smoking was associated with HbA1c levels (coefficient 0.021, 95% CI 0.003–0.039, *p* = 0.019). (Table 3)

In the logistic regression analysis for ≥ 5.7% HbA1c levels, the current cutoff value for abnormal glucose regulation, using the above-mentioned variables as covariates, age, gender, heavy alcohol drinking, waist circumference, smoking habits, hematocrit, and FPG were associated with HbA1c levels. Using never smokers as a control, current light smoking (OR 1.29, 95% CI 1.04–1.60, *p* = 0.002) and current heavy smoking (OR 1.84, 95% CI 1.41–2.41, *p* < 0.001) were associated with ≥5.7% HbA1c levels. Ex smoking was not associated with ≥5.7% HbA1c levels. Furthermore, the OR for an HbA1c levels ≥6.1%, a FPG level of 126 mg/dl, and a 2-h plasma glucose level ≥200 mg/dl, according to an oral glucose tolerance test, in Korea[21], was 2.36 (95% CI 1.58–3.52, *p* < 0.001) in current heavy smokers. In addition, former smokers showed no significantly increased risk of elevated HbA1c levels compared with never smokers. (Table 4)

Since the definition of diabetes did not include HbA1c level criteria in this study, 215 patients who had HbA1c levels ≥ 6.5% and FPG <126 mg/dL were included in study population. Therefore, we performed additional logistic regression for HbA1c ≥ 5.7% or ≥ 6.1% using above- mentioned variables as covariates, after excluding subjects with HbA1c levels ≥ 6.5%. Using never smokers as a control, current light smoking (OR 1.29, 95% CI 1.04–1.61, *p* = 0.023) and current heavy smoking (OR 1.83, 95% CI 1.39–2.41, *p* < 0.001) were associated with HbA1c levels ≥5.7%. (Table 5)

## Discussion

In the present study using KNHANES 2011–2012 data, we observed a significant association between smoking habits and HbA1c levels in the general adult population of Korea. Current smokers had higher HbA1c levels than former and never smokers in an exposure-dependent manner, even after adjusting for several clinical parameters that might affect the result.

Previously, an association between smoking and HbA1c levels was reported among healthy subjects without diabetes. Lincoln *et al*. discovered that mean HbA1c levels were lowest in never smokers, intermediate in former smokers, and highest in current smokers in a European multicenter International cohort study (the EPIC-Norfolk study) [15]. Their study also revealed an inverse association between years since smoking cessation and HbA1c levels. Jansen *et al*. also reported that smoking was independently associated with HbA1c levels in non-diabetic Dutch adults [16]. In the Scottish Health Survey, smokers had higher Hba1c levels than did non-smokers and were twice as likely to have HbA1c levels in the pre-diabetic range (5.7–6.4%) [22]. In a representative sample of the non-diabetic United States population, smokers had a 7% increase in HbA1c levels relative to never smokers[14].

All of the above studies were performed mainly in European and United States populations, which are both predominantly Caucasian. Furthermore, a recent meta-analysis revealed that higher HbA1c levels among current smokers are consistent in only Western population studies [17].

Nevertheless, this large population study of a racially homogeneous Korean population suggested that the association between smoking habits and HbA1c is also identifiable in non-Western populations without diabetes.

There are several hypotheses regarding the mechanism by which smoking increases HbA1c levels. Using data from the United States NHANES, Calore *et al*. reported that cotinine is associated with increased HbA1c levels [14]. Their study suggested that the link between smoking and HbA1c is at least partly due to the effects of nicotine. Consistent with this, a previous report showed that nicotine increased mTOR/P70S6K activity in cultured L6 myotubes, which was associated with increased IRS-1 Ser-636 phosphorylation, reduced insulin-stimulated glucose uptake, and subsequent insulin resistance [23]. Eliasson *et al*. found that the long-term use of nicotine-containing gum was associated with insulin resistance and hyperinsulinemia in subjects without diabetes [24]. However, in another study using euglycemic-clamps, nicotine infusion affected serum insulin levels in diabetic, but not healthy, subjects [25]. Nicotine also increased islet β-cell apoptosis via nicotinic acetylcholine receptors, leading to decreased insulin secretion. Mitochondrial dysfunction, oxidative stress, and inflammation all play a role in the direct toxicity induced by nicotine [26]. Taken together, these data suggest that nicotine exposure could negatively affect glucose metabolism by direct and/or indirect effects on insulin action, resulting in higher HbA1c levels even in non-diabetic smokers compared with never and former smokers.

Another hypothesis is that smoking could influence the formation of HbA1c indirectly, independent of its effect on blood glucose. Smoking might increase the passage of glucose across the erythrocyte membrane into cells, resulting in elevated HbA1c levels [27]. Soraya *et al*. suggested that smoking causes higher erythrocyte 2,3-diphosphoglycerate concentrations, conditions under which HbA1c formation is increased [17].

In addition, smoking causes increased oxidative stress and an associated increase in protein glycation, which can also increase HbA1c levels [22,28]. Conversely, antioxidant consumption and the levels of plasma antioxidants, such as vitamin C, were inversely correlated with HbA1c levels in non-diabetic individuals [29–31].

Although many clinical and experimental studies have reported an association between smoking and diabetes, it is unclear why smoking affects HbA1c levels, particularly in non-diabetic subjects. We excluded diabetic subjects in this study because use of anti-diabetes medications could have an effect on FPG and HbA1c levels. Furthermore, adjusting for confounding factors such as FPG had no effect on the statistical significance of the association between smoking and HbA1c. This supports the hypothesis that technical issues are associated with the HbA1c assay in samples from smokers, regardless of the mechanism involving smoking-induced glucose dysregulation.

In this study, we identified many non-glycemic determinants of HbA1c levels in non-diabetic subjects, in addition to smoking. The age-related increases in HbA1c levels reported in the current study were consistent with reports in large populations from China, Japan, and the United States [27,32–34]. These studies suggested altered rates of glycation and aging-associated renal function as possible explanations of age-related increases in HbA1c levels.

Alcohol consumption, a lifestyle parameter as important as smoking, was independently and negatively correlated with HbA1c levels, consistent with previous reports [29,35,36]. Previous studies suggested that moderate alcohol intake might have protective effects on glucose metabolism by lowering insulin resistance [37,38]. A meta-analysis also showed that moderate alcohol consumption decreased the relative risk for type 2 diabetes, compared to non-drinkers [39]. However, this positive effect of alcohol on glucose metabolism was attenuated in heavy drinkers, as heavy alcohol intake increased the risk of type 2 diabetes, compared to moderate alcohol intake. Such results suggest a “U-shaped” relationship between alcohol consumption and the risk of type 2 diabetes [39,40]. In the current study, the definition of heavy alcohol drinking was four or more drinks per week, which was less specific and more sensitive as a descriptor of high alcohol intake than the criteria of three or more drinks per day used in most previous studies [40]. Therefore, the correlation of heavy alcohol drinking with HbA1c levels between this and previous studies cannot be compared directly.

Weight circumference was also independently associated with HbA1c levels in this study. Because we included subjects with impaired FPG, some of them might have metabolic syndrome, which commonly includes central obesity and impaired FPG. Therefore, it is possible that there might be a residual confounding effect rather than a direct causal relationship.

We found that heavy smokers had higher BMI than current light smokers, never smokers, and ex-smokers, which is a contrast to the well documented effect for cigarette smokers to have lower BMI than non-smokers [41]. We speculate that this is due to clustering of risk behaviors potentially favoring weight gain in heavy smokers (e.g. unhealthy diet, physical inactivity) may outweigh the metabolic and anorectic effects of smoking. There are some studies showing similar results to our study. The Greek EPIC cohort study showed that tobacco smoking was positively associated with BMI and waist-to- hip ratio among smokers [42]. In the Swiss Healthy Survey, obesity was associated in a graded manner with the number of cigarettes smoked daily, particularly in men [43]. Another study also reported that moderate smokers had a decreased risk of obesity, compared with non-smokers (adjusted OR 0.4), but heavy smokers tended to have increased risk (OR 1.3) in Danish men 20 to 29 years of age [44]. Actually, heavy tobacco exposure exhibited the largest odds ratio (OR 2.4) for physical inactivity in the Danish study. Therefore, unhealthy life styles such as physical inactivity might account for the association between weight and smoking, especially in heavy smokers. The major strength of this study is that we included a large number of Korean adults and analyzed a nationally representative sample of adult Koreans without diabetes. To our knowledge, this is the first nationwide study that revealed a significant association between smoking habits and HbA1c levels in an Asian population. Although the difference in HbA1c between heavy smokers (5.60 ± 0.02%) and never smokers (5.52 ± 0.01%) was small in multivariable analysis, subjects with HbA1c levels around 5.7% (the current cutoff value for abnormal glucose regulation criteria) could be categorized as being at high risk for developing diabetes, depending on smoking status. The data showed that heavy current smokers were at a 1.83 times increased odds of belonging to the high risk group for developing diabetes, compared with non-smokers. Since this study included only subjects without diabetes, we could not confirm the effect of smoking status on diagnosis of diabetes with HbA1c of 6.5%. Nevertheless, this result suggested that smoking status needs to be taken into account when using HbA1c levels to screen for prediabetes or diabetes. In this respect, our findings hold clinical importance from a public health point of view.

This study has some limitations. First, we could not assess the accuracy of smoking exposure, because this study was based on self-reported smoking habits. Second, although we adjusted for many confounding factors, residual or hidden confounding variables could not be excluded, similar to other observational studies. We also could not draw an inference of causality due to the cross-sectional design of the study.

In conclusion, current smoking was independently associated with increased levels of HbA1c in a cigarette exposure-dependent manner in a nationally representative sample of Korean non-diabetic individuals. In this study, we observed an association between smoking status and HbA1c levels in non-diabetics from a non-Western population, consistent with previous findings in Western populations.
